# Magnesium deficiency and oxidative stress: an update

**DOI:** 10.7603/s40681-016-0020-6

**Published:** 2016-11-17

**Authors:** Anastasia A. Zheltova, Maria V. Kharitonova, Igor N. Iezhitsa, Alexander A. Spasov

**Affiliations:** 1 Department of Pharmacology, Volgograd State Medical University, Pl. Pavshikh Bortsov, 1, Volgograd, 400131 Russia; 2 Department of Immunology and Allergology, Volgograd State Medical University, Pl. Pavshikh Bortsov, 1, Volgograd, 400131 Russia; 3 Institute of Pharmacy, Department of Pharmacology and Toxicology, University of Innsbruck, Center for Chemistry and Biomedicine, Innrain 80-82/III, A-6020, Innsbruck, Austria; 4 Centre for Neuroscience Research (NeuRon), Faculty of Medicine, Universiti Teknologi MARA (UiTM), Sungai Buloh Campus, Jalan Hospital, 47000 Sungai Buloh, Selangor Darul Ehsan Malaysia; 5 RIG “Molecular Pharmacology and Advanced Therapeutics”, Pharmaceutical & Life Sciences (PLS) Communities of Research (CoRe),, Universiti Teknologi MARA, 40450 Shah Alam, Selangor Darul Ehsan Malaysia; 6 Faculty of Medicine, Sungai Buloh Campus, Jalan Hospital, Universiti Teknologi MARA, 47000 Sungai Buloh, Selangor Darul Ehsan Malaysia

**Keywords:** Magnesium, Magnesium deficiency, Oxidative stress, Antioxidants, Reactive oxygen species, Lipid peroxidation

## Abstract

Magnesium deficiency (MgD) has been shown to impact numerous biological processes at the cellular and molecular levels. In the present review, we discuss the relationship between MgD and oxidative stress (OS). MgD is accompanied by increased levels of OS markers such as lipid, protein and DNA oxidative modification products. Additionally, a relationship was detected between MgD and a weakened antioxidant defence. Different mechanisms associated with MgD are involved in the development and maintenance of OS. These mechanisms include systemic reactions such as inflammation and endothelial dysfunction, as well as changes at the cellular level, such as mitochondrial dysfunction and excessive fatty acid production.

## 1. Introduction

It is firmly established that deficiencies of essential macro- and micronutrients are associated with the development of different diseases [1-3]. However, the pathological consequences of a nutrient deficiency often lack a clear or direct relationship with the functions of that nutrient in the body. Magnesium deficiency (MgD) is an excellent example of this scenario [4].

MgD can be caused by numerous factors including decreased dietary Mg intake, stress [5], high levels of alcohol consumption [6], and inherited renal magnesium transport disorders [7] that are associated with excessive Mg loss. Additionally, endocrine diseases (diabetes mellitus [8], metabolic syndrome [9]) and administration of some medical agents (diuretics, proton-pump inhibitors, cardiac glycosides, epidermal growth factor receptor inhibitors, calcineurin inhibitors [10], aminoglycoside antibiotics, amphotericin B, cisplatin, pentamidine, and cyclosporine [11]) can also result in MgD. Several review articles have been published on Mg metabolism and related disorders [12, 13].

Prior literature, particularly studies using animal models, suggests a correlation between MgD and the development of oxidative stress (OS) [14]. However, Mg is not an acknowledged functional component of the antioxidant defence system (AOS).

Therefore, mechanisms of OS associated with a lack of Mg are still a matter of debate. Furthermore, the role of Mg in oxidative damage to molecules, cells and tissues in the pathogenesis associated with MgD remains unclear. Here, we present a critical analysis of the relationship between OS and MgD and a mechanism explaining the interaction between them.

## 2. Origin and measurement of oxidative stress

Sies H. defined OS in the body as “an imbalance between oxidants and antioxidants in favour of the oxidants, potentially leading to damage” [15]. The above-mentioned oxidants are ‘reactive species’ (RS) (reactive oxygen (ROS)/nitrogen/chlorine) [16]; some RS are free radicals. Many RS play a critical physiological role, and their production is essential for the normal life cycle of an organism. However, overproduction of RS can cause oxidative damage to molecules, cells and tissues [17], which contributes to the development of many diseases [18-20].

OS can be assessed by measuring an imbalance between RS oxidant production and the functional activity of the AOS. Both the production and the damaging activity of RS are complicated and multifaceted. Additionally, there are many components in the AOS interact to regulate OS [21]. Therefore, several different markers are used to measure the production of RS oxidants and the ability of the AOS to detect OS [16, 22]. Many authors speculate that an imbalance between pro- and anti-oxidants results in an increased level of oxidative degradation of biomolecule products, such as lipid peroxidation products. Furthermore, an increased concentration of oxidative damage markers can also indicate OS [23]. A wide range of available analytical approaches allows the quantification of lipid peroxidation and free radical-based DNA or protein damage [24]. However, many of these techniques lack sensitivity or specificity, particularly when estimating oxidant stress levels *in vivo*. Currently, there is no gold standard for measuring OS, *i.e*., a specific marker whose level is consistently affected by OS of different origins [25]. A recently started multiinvestigator project (the Biomarkers of Oxidative Stress Study (BOSS)) aims provide such a marker^1^; however, currently none of the existing methods for OS detection can be considered absolutely reliable.

## 3. Oxidative stress and magnesium deficiency

Early clinical studies have provided evidence of the impact of the OS associated with MgD on human pathology. The gold standard for verifying MgD in clinical studies is the parenteral Mg tolerance test (low dose Mg load test) [26, 27]. Unfortunately, this test was rarely used in published studies; thus, there is a lack of reliable clinical data that provides evidence for the relationship between MgD and OS.

Diabetic patients only displayed an increased concentration of oxidised LDL in association with a reduced level of serum Mg. Patients with normal serum Mg levels did not demonstrate this increase in oxidised LDL concentrations [28]. It has been shown that low dietary Mg intake is accompanied by poor DNA repair capacity [29] and increased genomic instability [30].

Barbagallo *et al*. established a strong, direct correlation between RBC Mg levels and GSH/GSSG concentration (circulating reduced/oxidized glutathione) (r = 0.84, *P* < 0.0001) [31]. In another study, a negative correlation between Mg levels and OS stress markers (plasma superoxide anions and malondialdehyde) was observed in groups of the population chronically exposed to stress [32]. Interestingly, no correlation between low Mg intake and antioxidant capacity has been found among Korean adults [33].

Animal studies were conducted to obtain biologically relevant evidence of causal relationships between MgD and OS. Mgdeficient feed is used to induce dietary MgD in animals. It was demonstrated that the lipoprotein fractions (VLDL and LDL) from three-week old Mg-deficient rats were more susceptible to oxidative damage caused by CuSO4-induced oxidation than lipoprotein fractions from control rats. The triacylglycerol and alphatocopherol levels in plasma were significantly higher, whereas the level of alpha-tocopherol in the VLDL + LDL fraction was significantly lower in the Mg-deficient group compared to the control group. After exposing tissue homogenates to Fe-induced lipid peroxidation, the concentration of thiobarbituric acid-reactive substances was significantly higher in tissues from Mg-deficient rats than in those from control rats [34].

MgD was accompanied by a two-fold decrease in glutathione (GSH) concentration in RBCs [35]. In other types of cells, the overexpression of glutathione transferase has been suggested to be the cause of GSH depletion [36]. After six weeks, the MgD diet led to a significant decrease of both plasma and RBC Mg levels, followed by a marked increase in plasma malondialdehyde and a corresponding decrease in the total number of radical-trapping antioxidant markers [37]. In another study, rats fed the MgD diet displayed an impaired redox capacity, marked by increased levels of thiobarbituric acid-reactive substances and oxysterols in the plasma as well as an increased susceptibility of RBC to freeradical- induced haemolysis *in vitro* [38]. High levels of thiobarbituric acid-reactive substances in the aorta of rats fed the Mg deficient diet correlated with a significant reduction in the activity of superoxide dismutase and catalase as well as an increase in the net fractional rates of collagen synthesis [39]. In mice, hypomagnesaemia led to a decrease in GSH concentration and lowered the activity of superoxide dismutase, glutathione reductase, and glutathione S-transferase in RBCs. However, catalase activity increased and the activity of glutathione peroxidise was not significantly altered [40]. Boparai *et al*. found evidence for lipid peroxidation and protein oxidation in the plasma and liver of rats that received a low Mg diet [41]. Based on these findings, we investigated the effects of MgD on the intensity of protein oxidative damage. Fifty adult, female Wistar rats with weights between 200-250 g were divided into two groups. One group received a low Mg diet (Mg content ≤ 15 mg/kg) and demineralized water for two months to induce hypomagnesaemia. The other group was fed a basal control diet (Mg content ≈ 500 mg/kg) and water (with Mg content 20 mg/l) for an equal duration. To evaluate the Mg concentration, a two ml sample of heparinized venous blood was collected every week from the sublingual vein while the rats were under isoflurane anaesthesia [42]. RBC and plasma Mg levels were measured *via* a previously described colorimetric assay method in which Mg is stained using thiazole yellow [43]. After Mg concentration in rats fed the low Mg diet had decreased, the animals were treated with one of the following supplementations: Mg L-aspartate, Mg N-acetyltaurate, Mg chloride or Mg sulphate (50 mg of elementary Mg per kg body weight for 14 days). Protein carbonyls (PC) concentration was assessed using the reaction of carbonyl groups with 2, 4-dinitrophenylhydrazine (DNPH) and measuring the resulting protein-bound 2, 4-dinitrophenylhydrazones. The yellow product was quantified by spectrophotometry at 363 nm [44]. We then calculated the ratio between the concentrations of carbonyl products (mol/L) and total protein (g/L). We found that the increased level of PC in rats fed the low Mg diet was partially or completely reversed by treatment with certain organic and inorganic Mg salts [45].

Some research teams have focused on understanding these mechanisms on the cellular level. MgD promoted apoptosis in rat hepatocyte primary culture, which was accompanied by an accumulation of malondialdehyde and a decreased GSH concentration [46]. N-acetylcysteine partially attenuated the apoptosis of human and rat hepatocytes induced by low extracellular Mg concentrations, but surprisingly, also increased both caspase-3 activity and lipid peroxidation in hepatocytes exposed to physiological Mg concentrations [47]. Two hours after exposure to low Mg, human umbilical vein endothelial cells (HUVEC) were more sensitive to the oxidant action of H_2_O_2_ and demonstrated an increased level of the DNA damage marker 8-hydroxy-deoxyguanine compared to controls cultured in physiologic concentrations of Mg [48]. Dickens *et al*. found enhanced free radical-induced intracellular oxidation and cytotoxicity in bovine endothelial cells incubated in a low-Mg medium [49].

**Fig. 1:**
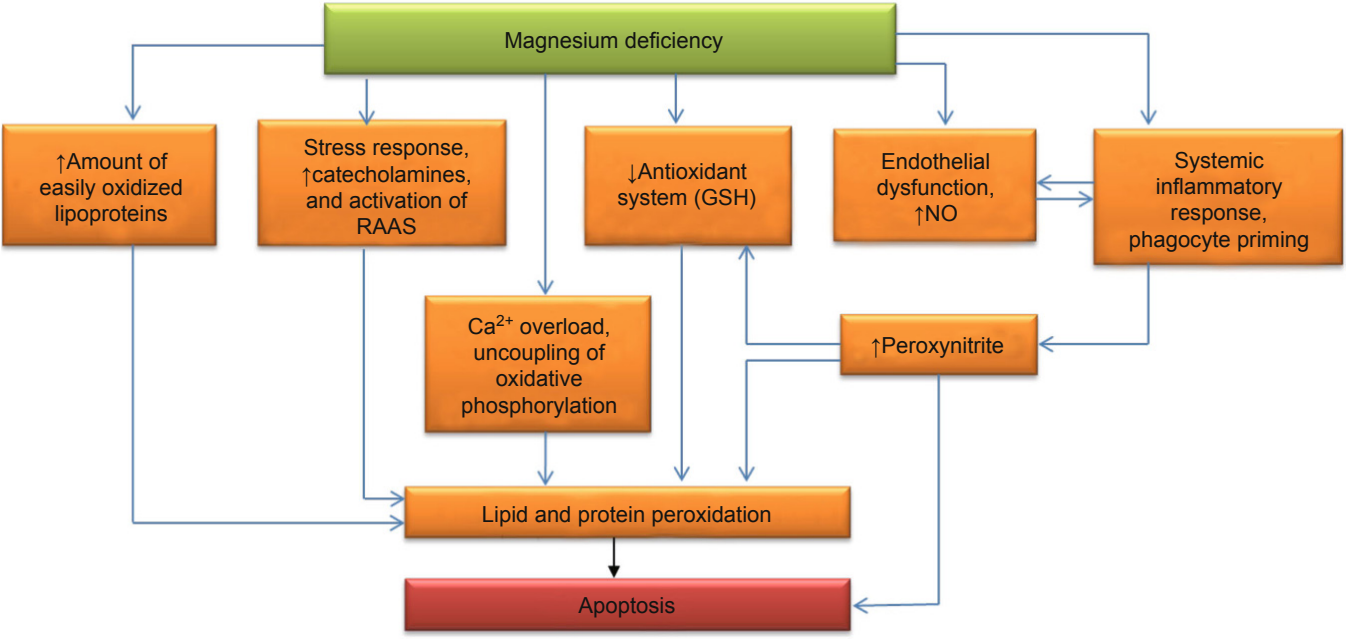
- Pathogenic relationship between magnesium deficiency and oxidative stress.

## 4. Mechanisms of oxidative stress caused by magnesium deficiency

MgD is believed to indirectly enhance oxidative damage of biomolecules by inducing a stress response (Figure 1). It is possible that a decreased ratio of Mg to Ca stimulates catecholamine release from the adrenal glands. However, catecholamines increase the production of ROS. This creates a vicious positive feedback cycle where, for example, elevated blood epinephrine levels result in a further reduction of the Mg concentration [50]. Contrastingly, MgD leads to the activation of the rennin-angiotensin system that also induces OS [51].

Inflammation is the other important cause of the OS that results from MgD [52]. MgD stimulates the production of acute phase proteins (*e.g.*, C-reactive protein) [53]. The decrease of extra- and intracellular Mg concentrations sensitizes immunocompetent cells to proinflammatory stimuli. Collectively, factors that would not normally cause an immune response lead to an oxidative burden in phagocytes and neutrophil activation in Mgdeficient organisms. Furthermore, low a blood Mg concentration directly stimulates phagocyte priming and results in oxidative burden [54], possibly due to the rise of intracellular Ca levels [55]. Excessive amounts of RS, created by NADPH oxidase and myeloperoxidase, enter into the space around the neutrophils and macrophages [55] and damage biomolecules, particularly components of lipoproteins and the surrounding cells [56]. In contrast, Mg repletion therapy promotes an anti-inflammatory response and decreased levels of proinflammatory markers in initially Mg deficient rats [57, 58].

Another early marker of MgD is endothelial dysfunction [59]. Under physiological conditions, the endothelium produces signalling molecules, which maintain the dynamic balance between thrombin formation and fibrinolysis. These signalling molecules also control and inhibit excessive synthesis of proinflammatory cytokines [60]. The endothelial dysfunction linked to MgD has one important feature. Endothelial dysfunction is frequently associated with reduced NO production in endotheliocytes [61]. However, preclinical studies in animal and tissue models have demonstrated that MgD actually increased NO production in the endothelium and other cells *via* the activation of an inducible isoform of NO-synthase [62-65]. Elevated NO production can be a disadvantage because it is accompanied by a simultaneous increase of RS, such as superoxide [66]. Under these conditions, excessive NO does not cause vasodilation, but rather, reacts with superoxide to form peroxynitrite [67]. A potent vasoconstrictor, peroxynitrite easily causes oxidative damage to biomolecules and cellular structures [68, 69]. Mak *et al.* have shown that, in particular, excessive NO production is responsible for a decreased concentration of GSH in red blood cells [62]. Moreover, hyperproduction of NO can provoke the apoptosis of certain cell types [70]. Finally, endothelial dysfunction and a hyperactivated inflammatory response can potentiate each other [61].

Intracellular production of RS can be enhanced by impaired mitochondrial function. MgD facilitates the uncoupling of oxidative phosphorylation, which leads to electron loss in the electron transport chain [71]. Low Mg levels result in an accumulation of calcium in the cytosol [72, 73] that contributes to the uncoupling of oxidative phosphorylation as well as the stimulation of other peroxidation pathways [74-76]. An overproduction of peroxynitrite that also results from MgD further exacerbates mitochondrial dysfunction [77, 78].

Apart from the enhanced generation of ROS and free radicals, MgD also increases the amount of substrates that are available for radical oxidation. MgD promotes hypertriglyceridemia, in which numerous, easily-oxidized lipoproteins enter the blood stream [79] and the activity of lipoprotein lipase is down-regulated [80]. Additionally, MgD contributes to insulin resistance and the overproduction of contra-insulin hormones (epinephrine and cortisol) [81, 82]. The key factors implicated in hyperlipidaemia are: the activation of lipolysis in fat tissue, the excessive release of free fatty acids, the stimulation of lipogenesis in the liver followed by the hyperproduction of triglyceride-rich atherogenic lipoproteins and the inhibition of HDL synthesis [34, 83-85]. In cellular membranes, an increased ratio of Ca to Mg stimulates phospholipase A2 activity [86, 87], which is responsible for the mobilisation of unsaturated fatty acids (UFA) from phospholipids. Free UFA as well as those bound to triglycerides and phospholipids can be easily oxidized by ROS to form lipid hydroperoxides. These hydroperoxides can decompose to form new radicals, thus initiating branching chain reactions that lead to a self-sustaining peroxidation process [88, 89].

## 5. Suggestions for clinical application

As Mg is suggested to be an important player in the pathogenesis of diseases [2-13, 90-93] and is associated with disturbed antioxidant regulation [28, 31, 32, 37-40, 45, 48-50], estimation and correction of impaired magnesium status is highly recommended in MgD patients.

## 6. Conclusion

To summarise, MgD and OS are undoubtedly strongly linked together. Moreover, several well-established and also several emerging mechanisms of OS in Mg deficient organisms were described. Nevertheless, many aspects of the causal relationship between MgD and OS still remain fragmented. Therefore, further preclinical and clinical studies are necessary to clarify the mechanisms involved in relationship between MgD, OS and OSassociated diseases.

## Footnotes

1
http://www.niehs.nih.gov/research/resources/databases/bosstudy/index.cfm

## Abbreviations:

Mg: Magnesium
MgD: Magnesium Deficiency
OS: Oxidative Stress
AOS: Antioxidant Defence System
RS/ROS: Reactive Species / Reactive Oxygen Species
BOSS: Biomarkers of Oxidative Stress Study
GSH/GSSG: Reduced/Oxidized Glutathione
RBC: Red Blood Cell
PC: Protein Carbonyls
DNPH: 2,4-Dinitrophenylhydrazine
HUVEC: Human Umbilical Vein Endothelial Cells
VLDL: Very-Low-Density Lipoprotein
LDL: Low-Density Lipoprotein
UFA: Unsaturated Fatty Acids
